# A Wearable Open-Source electrical impedance tomography device

**DOI:** 10.1016/j.ohx.2024.e00521

**Published:** 2024-03-20

**Authors:** Andrew Creegan, Joshua Bradfield, Samuel Richardson, Llewellyn Sims Johns, Kelly Burrowes, Haribalan Kumar, Poul M.F. Nielsen, Merryn H. Tawhai

**Affiliations:** a Auckland Bioengineering Institute, The University of Auckland, Auckland 1010, New Zealand; b Department of Engineering Science, Faculty of Engineering, The University of Auckland, Auckland 1010, New Zealand

**Keywords:** Electrical impedance tomography, EIT, Medical imaging, Open source, Wearable

## Abstract

Electrical impedance tomography (EIT) is medical imaging technique in which small electrical signals are used to map the electrical impedance distribution within the body. It is safe and non-invasive, which make it attractive for use in continuous monitoring or outpatient applications, but the high cost of commercial devices is an impediment to its adoption. Over the last 10 years, many research groups have developed their own EIT devices, but few designs for open-source EIT hardware are available. In this work, we present a complete open-source EIT system that is designed to be suitable for monitoring the lungs of free breathing subjects. The device is low-cost, wearable, and is designed to comply with the industry accepted safety standard for EIT. The device has been tested in two regimes: Firstly in terms of measurement uncertainty as a voltage measurement system, and secondly against a set of measures that have been proposed specifically for EIT hardware. The voltage measurement uncertainty of the device was measured to be − 0.7 % ± 0.36 mV. The EIT specific performance was measured in a phantom test designed to be as physiologically representative as practicable, and the device performed similarly to other published devices. This work will contribute to increased accessibility of EIT for study and will contribute to consensus on testing methodology for EIT devices.

## Graphical abstract

**Figure:**
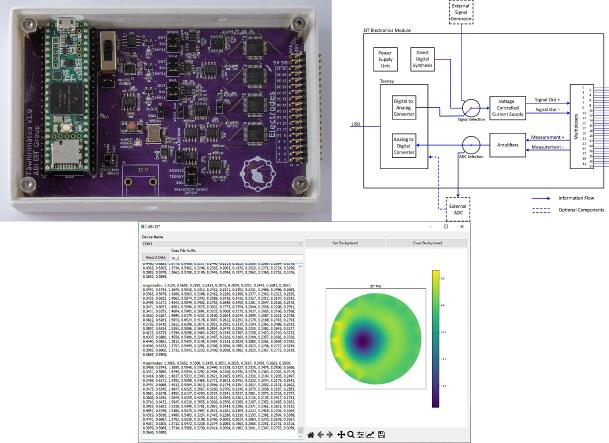

## Specifications Table

**Table:** 

Hardware Name	ABI EIT
Subject Area	Medical imaging
Hardware Type	Imaging tools
Closest commercial analog	Spectra (Mindseye Biomedical)
Open source license	GPL V3.0
Cost of hardware	260 USD
Source file repository	https://doi.org/10.17632/xn4pj3rt7b.1

## Hardware In Context

Electrical impedance tomography (EIT) is a technique in which the interior of the human body can be imaged using small electrical signals. In EIT, electrodes are arranged around the exterior of the body, and tiny electrical currents are applied across several of them, while the voltages are measured at the remainder. Using a tomographic reconstruction algorithm, an image of the electrical impedance distribution within the body can be created [1]. EIT has been proposed for many medical applications, including for monitoring lung function, cardiac function, brain activity, and others [1]. In the application of lung monitoring, it is particularly useful since the air in the lungs is strongly correlated with electrical impedance[2], resulting in relatively high contrast images. EIT is safe and non-invasive, which makes it an attractive technology for continuous monitoring of lung health. Despite these benefits, it is not yet in widespread use. The path to wider adoption of EIT is multifaceted, but two major challenges are in its relatively high cost and the requirement for sophisticated algorithms to produce useful images [3]. For example, despite the relatively low hardware requirements for an EIT device, one of the standard commercial EIT systems (Drager PulmoVista500) costs 25,000 USD to 39,000 USD [4] (Note that these prices, while expensive for bedside monitoring devices, are still much lower than for some alternative scanning technologies, such as CT or MRI machines). An effective, low-cost EIT system could help make routine EIT monitoring more achievable.

Over the last 10 years, many research groups have developed their own EIT devices [5], [6], [7], [8], [9], [10], [11], [12], [13], [14], [15], [16], [17], most being low cost and wearable. Unfortunately, few of these groups have made their schematics publicly available, and few such low-cost EIT devices are available commercially. Table 1 compares some of the existing EIT devices.

**Table 1:** Comparison of EIT devices.

Device	Availability	Cost	No. Electrodes	Excitation Frequency	Frame Rate	Safety	Form Factor
PulmoVista 500 (Dräger)	Commercial	$25,000 to $39,000 USD	16	80 to 100 kHz	Up to 50 Hz	IEC 60,601	Free standing
Sheffield Mark 3.5 (Maltron)	Commercial	–	8	2 to 1620 kHz	25 Hz	IEC 60,601	Free standing
Spectra (Mindseye Biomedical)	Commercial	$1,295 USD	8–32	80 Hz to 80 kHz	0.2 Hz	IEC 60,601	Wearable
Tomo 2 (CMU) [6]	None	Low (Common components)	8–32	40 kHz	6–100	No standard discussed	Wearable
NUAA [7]	None	Approx $400	8	100 kHz	–	No standard Discussed	Multi PCB
KAIST [8]	None	Unknown (Custom IC)	32	10 to 200 kHz	20	No standard discussed	Wearable
STUST [11]	None	Low (Common components)	16	10 to 200 kHz	–	No standard discussed	Wearable
University of Montpelier [14]	Open Source	$270 USD plus external equipment	16	1 to 50 kHz	–	No standard discussed	Multiple benchtop units
ScouseTom (UCL) [15]	Open Source	Unknown (Common components plus external equipment)	Up to 256	20 Hz to 20 kHz	> 100	IEC 60,601	Multiple benchtop units
University of Cauca (Muñoz et al.) [18]	Open Source	80.5 USD	8	50 kHz	100 Hz	IEC 60,601	Wearable
University of Cauca (Leyton et al.) [19]	Open Source	49.17 USD	32 (4 rings of 8)	50 kHz	50 Hz	IEC 60,601	Wearable
This work	Open Source	260 USD	16–32	25 kHz	Up to 25 Hz	IEC 60,601	Wearable

In contrast to the commercial devices that are available, which tend to be large, free standing units for use in hospitals, most research devices are designed to be wearable and low cost. Many use similar circuit topologies, such as Howland current pumps, multiplexers, instrumentation amplifiers, and single board microcontrollers. This tends to place them on similar footing in terms of size, cost, and performance. Two groups have made some EIT hardware designs open-source [14], [15], but neither are a complete system, relying on external benchtop signal generators, analogue to digital converters (ADCs), etc.

Most EIT devices feature 8, 16, or 32 electrodes, with 16 being the most common configuration in practice, balancing scan time with information gathered. Typically, excitation frequency (i.e., frequency of the injected current) varies from 10 kHz to 200 kHz, but most research groups do not attempt multi-frequency EIT, which has been said to require 800 kHz as a minimum upper limit for tissue characterization [20].

Very few publications of EIT devices discuss safety of the device for use on human subjects, even though most are intended for use on humans, often placed across the chest. The accepted safety standard for commercial EIT devices, IEC 60601, limits AC current across a subject to 100 µA. Some published devices use currents far higher than this, for example Tomo 2, at 300 µA [6], or the KAIST system, at 400 µA [8].

Two recently published articles from the university of Cauca propose a more complete open source systems, but feature, respectively, 8 electrodes[18], and 4 rings of 8 electrodes [19] limiting the achievable in-plane imaging resolution.

The lack of a suitable complete open-source EIT device motivated us to develop and release the system proposed in this work. It is composed of a low cost, portable, electronics module along with a desktop application which can perform real-time reconstruction on a tablet computer such as a Microsoft Surface. Firmware on the electronics module automatically controls signal generation, addressing of excitation and measurement from up to 32 electrodes, measurement amplification, and demodulation. The resulting boundary measurements are streamed to the PC in real time over a USB connection.

The specifications for the proposed device were driven by the research interests of our group. We are interested in lung monitoring, particularly on a continuous basis in an outpatient setting. We therefore required a low-cost, portable device designed for human safety, and capable of imaging free-breathing subjects. In this paper, an EIT system that meets these requirements is proposed. All schematics, firmware, and software have been made available with an open-source license.

Comparison of the performance of EIT devices is difficult because no consensus has been adopted on how performance should be measured. Most publications attempt to measure device performance in terms of a specific application, which can be dependent on a large number of factors, including intrinsic device characteristics, but also algorithms, selected operational parameters, and test conditions [11], [12], [15], [6], [7], [8]. In this paper, device performance is assessed in two ways: 1) Measurement accuracy is reported in terms generic to measurement systems, i.e., as statistical uncertainty of individual scalar measurements compared to an accepted reference, taken under specified conditions. (2) The performance of the device as an EIT system specifically is assessed according to the measures proposed by *Yasin et al (2011)* [21].

## Hardware Description

**Fig. 1:**
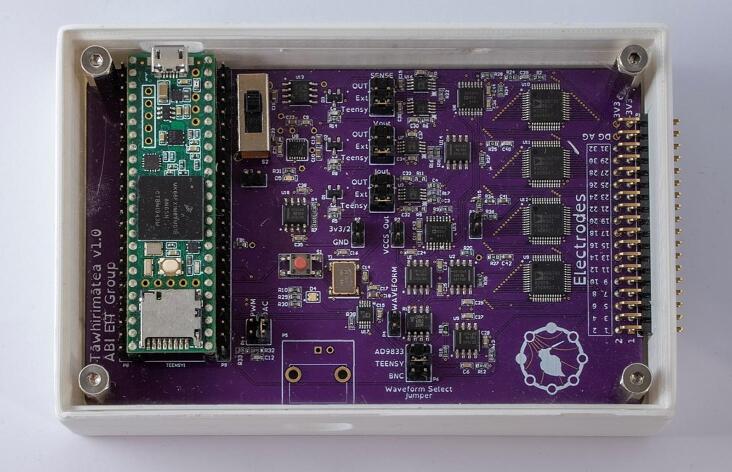
The electronics module of the EIT device.

The system is comprised of three sections: electronics, firmware, and user interface software. The electronics section is a single PCB responsible for generating the excitation signal and measuring the resultant voltages. Data is processed by the onboard microcontroller and output via a serial connection to a PC. The firmware is the code running on the microcontroller. It controls the excitation waveform and measurement electronics, processes the measurements, and manages serial communications. The user interface software is a desktop application that makes use of the pyEIT library [22] for real time reconstruction, as well as allowing the user to save data. Fig. 1 shows the electronics module enclosed in a 3D printed case.

The specifications of the system are as shown in Table 2.

**Table 2:** EIT device specifications.

Parameter	Value
Excitation Frequency	25 kHz
Output Current	98 µA
Number of Electrodes	4–32
Physical Dimensions	120 mm x 80 mm x 32 mm
Frame Processing Rate	25 Hz
Voltage Measurement Uncertainty	−0.7 % ± 0.36 mV
Cost	262 USD

### Electronics

**Fig. 2:**
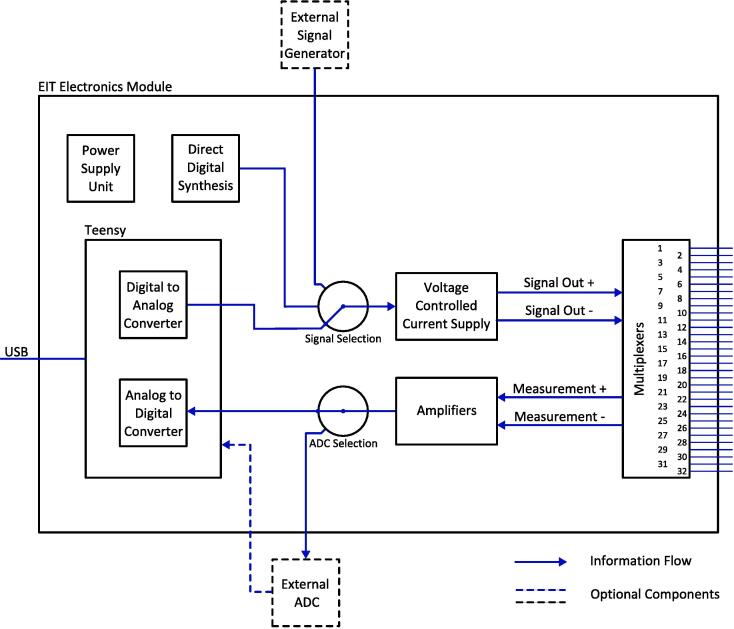
Block diagram of the EIT electronics module.

Fig. 2 is a module level diagram of the EIT electronics module. The functionality begins with the Teensy microcontroller [23], where a sinusoidal voltage signal is generated using the digital to analogue converter. The signal is converted to a current in the voltage controlled current supply module. The positive and negative terminals of the current signal are each connected via the multiplexer module to one of the 32 electrodes. The multiplexer module also connects two of the 32 electrodes to the measurement amplifier module. Amplified signals are then fed back into the Teensy via the analogue to digital converter. Communications to and from the external PC are made with the Teensy’s USB serial device.

### Teensy

A Teensy 3.6 from PJRC was selected for the EIT electronics module because of its low cost and on-board ADC, and digital to analogue converter (DAC). At the time of writing, the listed price of the Teensy 3.6 is 29.25 USD. Its processor is an ARM Cortex-M4 rated at 180 MHz It has an on-board DAC with 12-bit resolution, and an on-board ADC with 16 bit resolution (PJRC advise that under normal conditions, the effective resolution of the ADC is 13 bit due to noise).

In the EIT electronics module, the Teensy performs the following tasks:

• Signal generation: The Teensy’s ADC is used to generate a voltage sine wave. By default, the full sine wave is comprised of 16 voltage levels, with a 0.6 V peak-to-peak (or 0.21 V RMS) and a frequency of approximately 25 kHz.
• Signal measurement: The Teensy’s digital to analogue converter (DAC) is used to digitize the board’s measurements so that they can be further processed.
• Demodulation: Amplitude and phase information are extracted from the discrete sampled voltages.
• Communication: Processed data is sent to the PC.

### Direct Digital Synthesis (DDS)

The DDS module provides the optional functionality of generating an alternative analogue waveform. The frequency can be controlled by the Teensy via the SPI bus. The magnitude of the waveform is 0.6 V peak-to-peak.

### Voltage Controlled Current Source (VCCS)

The VCCS is the most complex circuit on the EIT electronics module. It generates a controlled current signal (a current of amplitude that oscillates sinusoidally at a specified frequency) using the voltage signal from the teensy as input. The current signal is sent to the EIT device output. A current controlled signal is required (instead of voltage) because safety standards limit current applied across the body. To achieve the highest possible signal to noise ratio, the largest safe amplitude drive signal should be used.

**Fig. 3:**
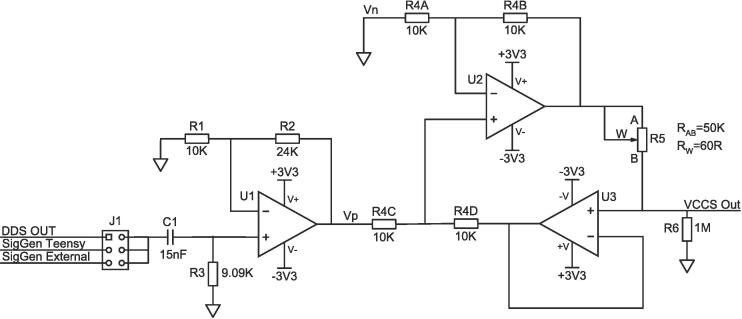
Voltage controlled current source (VCCS) circuit diagram.

The circuit used for the VCCS is known as a modified Howland current pump [24]. It is shown in Fig. 3. The operation is as follows:

Input is selectable from one of three sources. DDS, Teensy, or an external signal generator. Selection is done using a jumper on connector J1. The input should be a 0.21 V RMS signal. C1 acts as a high pass filter which removes the DC offset from the signal so it can be used to drive the differential supply Op-amps in the circuit (U1, U2, and U3).

Prior to the Howland circuit proper, a non-inverting amplifier circuit is formed using U1. The gain of this amplifier is:

(1) $$A_{U1}=1+\frac{R2}{R1}=3.4$$

This gain was selected to reduce noise and quantization error, while still allowing the designed current output of the VCCS.

The Howland circuit is composed of the amplifiers U2 and U3. It generates a current proportional to the input voltage:

(2) $$I_{\mathrm{VCCSOut}}=\frac{A_{\mathrm{Howland}}\times \left(V_{p}-V_{n}\right)}{R5},$$

where the Howland gain $A_{\mathrm{Howland}}$ is determined by the resistors R4A, R4B, R4C, and R4D:

(3) $$A_{\mathrm{Howland}}=\frac{R4B}{R4A},given:R4A=R4CandR4B=R4D$$

To ensure matched resistance between R4A and R4C, and between R4B and R4D, a matched resistor grid is used. The same grid is used for all four resistances, resulting in a gain of 1:

(4) $$A_{\mathrm{Howland}}=1,given:R4A=R4C=R4B=R4D$$

With $V_{n}=0$, and $V_{p}=SigGenTeensy\times A_{U1}=0.72$, we have:

(5) $$I_{\mathrm{VCCSOut}}=\frac{0.72}{R5}.$$

R5 is a digital potentiometer connected to the Teensy via SPI, allowing the VCCS to be accurately controlled with a single variable in firmware.

### Multiplexers

The EIT electronics module multiplexers make connections from inputs and outputs to all external pins. There are two output terminals (Signal Out + and Signal Out -) and two input terminals (Measurement + and Measurement -). For each individual measurement, these four terminals are each connected to one of the 32 external pins. During an EIT measurement frame, each of the external pins connected to the measurement subject acts as both input and output. See the section *Excitation Matrix* below for more details on this process.

### Amplifiers

The amplifiers are responsible for amplifying the voltages at the boundary of the measurement subject to levels appropriate for the Teensy ADC. The EIT electronics module was designed with three sets of amplifiers for three different measurements: input voltage (i.e., voltage measurements used for EIT), output voltage (i.e., the voltage between the signal terminals of the device), and output current (i.e., the current level of the signal). Presently, only the input voltage amplifier circuit is used.

**Fig. 4:**
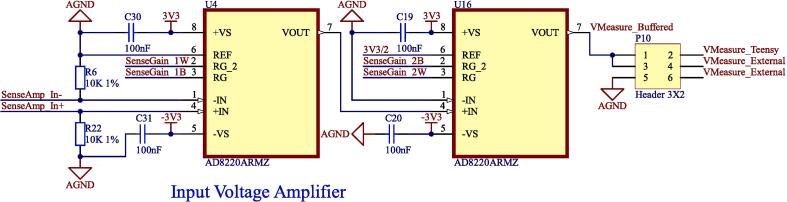
Input voltage amplifier.

The input voltage amplifier circuit is a two-stage differential amplifier. Both stages have digitally controllable gain using digital potentiometers. Fig. 4 shows a diagram of the input voltage amplifier circuit.

Both amplifier stages use the Analog Devices AD8220ARMZ instrumentation amplifier. This device has the gain equation:

(6) $$G=1+\frac{49.4k\Omega }{R_{G}},$$

where $R_{G}$ is a resistor connected to the device gain terminals. The digital potentiometer model is AD5262BRUZ50, with a wiper resistance of less than 150 Ω, and a maximum resistance of 50 kΩ.

The total gain of the two stages is:

$$G_{\mathrm{Total}}=\left(1+\frac{49.4k\Omega }{R_{G1}}\right)\times \left(1+\frac{49.4k\Omega }{R_{G2}}\right)$$

Giving a total minimum gain of 4 and a total maximum gain of at least 100,000.

### Device Safety (IEC 60601)

The proposed device is designed to be compliant with the IEC 60601 standard on medical electrical equipment. In particular, the circuit complies with the allowable patient auxiliary currents as specified in the standard. Table 3 below taken is from the standard.

**Table 3:** Maximum current levels for device safety.

		Type BF Applied Part	
		Normal Condition	Single Fault Condition
Patient Auxiliary Current (µA)	DC	10	50
	AC	100	500

**Fig. 5:**
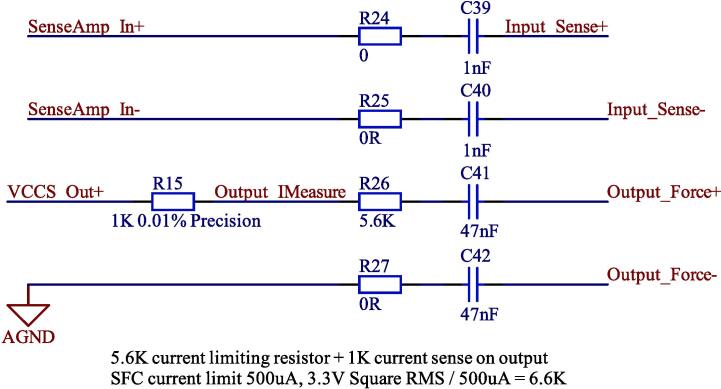
Input and output terminals.

Fig. 5 shows the input and output terminals of the electronics module. A decoupling capacitor on each terminal prevents any DC current from entering the measurement subject. Current limiting resistors on the output terminals keep AC current at safe levels during a single fault condition.

In the normal condition, the DC current is 0. In the single fault condition, the DC current is also 0 because there are series capacitors attached to all connections to the human body in the circuit.

The AC current is controlled by the circuit to be below 100 µA in the normal condition. In the single fault condition, the maximum AC voltage will be a square waveform oscillating between the positive and negative supply voltages. Under this condition, the current is held below 500 µA using a series resistor at the output of the excitation subcircuit:

(8) $$V_{p-p}=6.6V$$

(9) $$R_{\mathrm{safety}}=5.6k\Omega +1k\Omega$$

(10) $$V_{\mathrm{rms}(square)}=0.5\times V_{\mathrm{pk}-pk}=3.3V$$

(11) $$I=\frac{V}{R}=500\mu A$$

## Firmware

### Firmware Overview

**Fig. 6:**
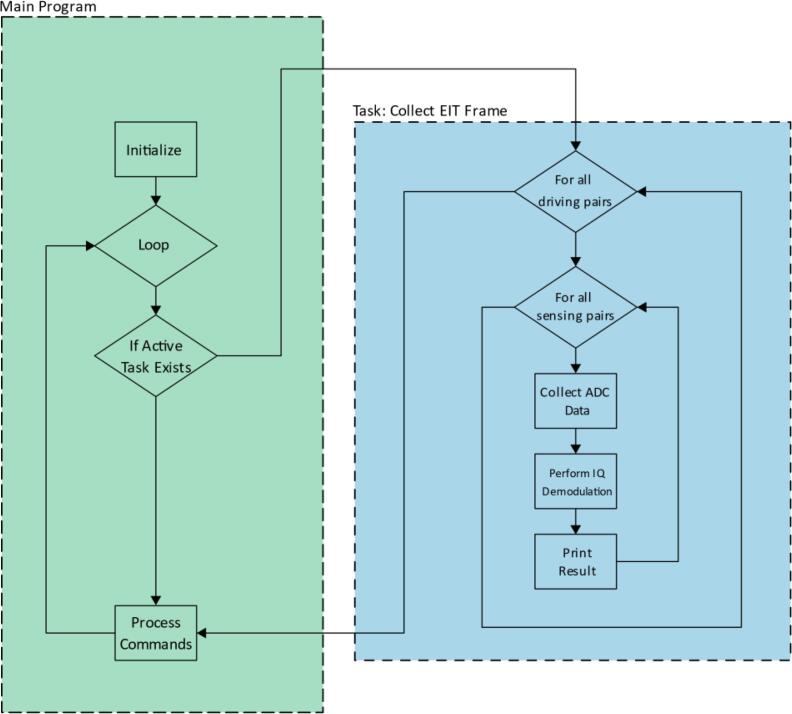
Block diagram of EIT firmware.

The firmware is written in C++ for the Teensy microcontroller. The firmware is open source and provided in supplementary material in the EIT-Device-Firmware.zip file. Fig. 6 is a top-level diagram of the program’s operation.

The operation is as follows:

### Initialize

The initialize phase runs once when the device is turned on. During this step, peripherals on the Teensy are initialized, such as the DAC, Serial, and ADC. The DAC is configured to continuously generate the output signal. Gain levels for the device amplifiers are set to default levels.

### Loop

The program then enters a continuous loop. Any task that it is configured to perform is executed on each iteration of the loop. By default, this is the “Collect EIT Frame” task.

In the Collect EIT Frame task all combinations of driving electrode pairs and sensing electrode pairs are addressed by the multiplexers, with a single EIT measurement being performed for each (see *Excitation Matrix* for details on this sequence). The EIT measurement is performed by first collecting a series of individual ADC measurements. These are processed (See *I/Q Demodulation*) to extract the amplitude of the sinusoidal signal before being printed to serial.

After the active task is executed, each loop of the main program ends by processing any commands received over serial. These commands allow the behaviour of the device to be changed, either by modifying variables such as amplifier gains, or setting a different active task.

### Excitation Matrix

**Fig. 7:**
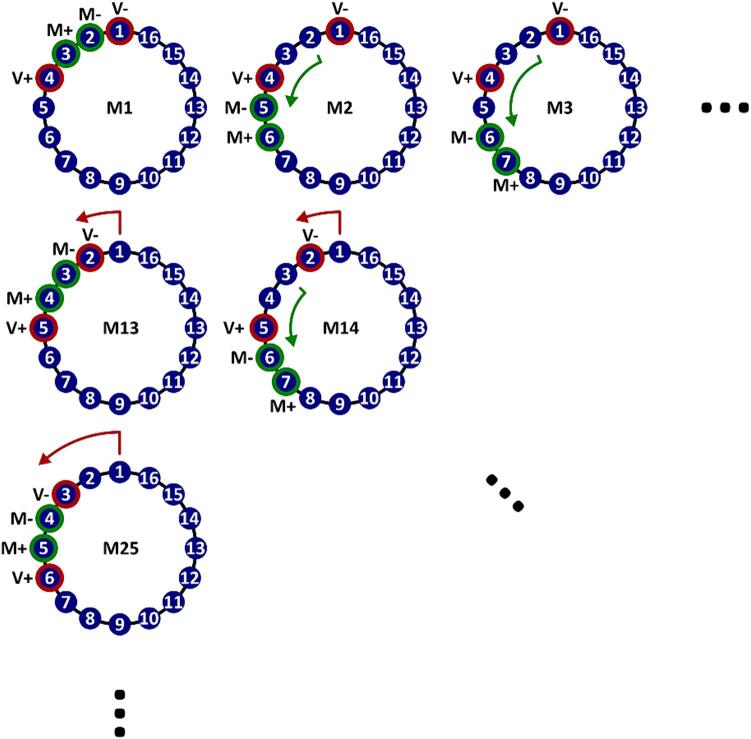
Excitation protocol.

The excitation matrix is the matrix generated by all combinations of excitation electrodes and measurement electrodes in an EIT frame. The protocol by which this matrix is constructed is illustrated in Fig. 7. The combinations of excitation and measurement electrodes are constructed as follows:

1. **Excitation Electrodes:** For the excitation electrodes (V + and V − ), starting at electrode 1, V + is set to the next highest electrode in sequence, and V- is set to an electrode of a fixed number higher than V +. This continues until all electrodes have been used as V +.
2. **Measurement Electrodes:** For each pair of excitation electrodes, the list of measurement electrodes (M + and M-) is generated with M- starting at the electrode one higher than V-, and M + set to an electrode a fixed number higher than M-. If either M + or M- is set to the same electrode as V+, the pair is skipped. M + and M- are increased until M- reaches V-.

Fig. 7, illustrates the protocol with the default settings: 16 electrodes, excitation electrodes separated by 3, and measurement electrodes separated by 1.

This excitation protocol corresponds to the matrix generated using the following pyEIT function:

protocol.create(n_electrodes = 16, dist_exc = 3, step_meas = 1, parser_meas = fmmu).

In this function, n_electrodes refers to the number of electrodes used. dist_exc refers to the distance in electrode indices between V + and V−, step_meas refers to the distance in electrode indices between electrodes M + and M-, and parser_meas determines whether subsequence measurements start from after V+ (as in this case) or from electrode 1. Note that the firmware has been configured with a default dist_exc parameter of 3, which avoids potential performance reduction from directly adjacent or oppositional excitation [25], but has not been tested to be optimal.

### Signal Sampling

**Fig. 8:**
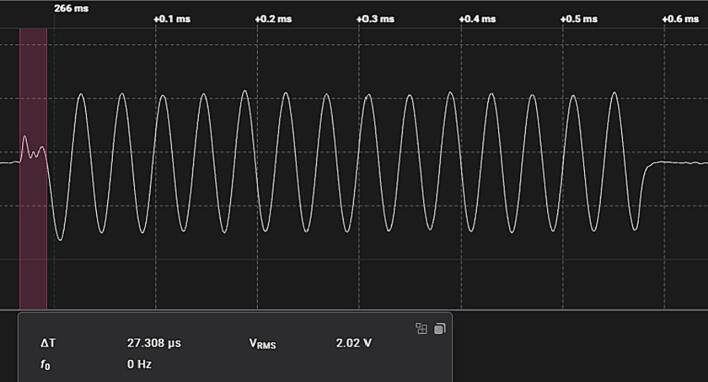
Amplified signal showing post-switching instability. Highlighted time period shown as ΔT.

At each point in the excitation matrix, the amplified signals are sampled by the ADC over a number of excitation signal cycles. Directly after switching, the signal is not stable, so the first few samples are discarded before processing the data. As shown in Fig. 8, the unstable period lasts for about 30 µs. Discarding 10 samples at a 25 kHz excitation frequency, sampling 10 times per cycle results in a period of 40 µs, sufficient time to avoid the instability.

### I/Q Demodulation

As the EIT device cycles through the excitation matrix, a number of discrete samples are collected at each position. These values represent discrete measurements of the resultant electrical signal across points A and B when the excitation is applied at points V + and V −. Before the EIT reconstruction can be carried out, the amplitude and phase of the continuous signal must be computed from the sampled values. A process known as I/Q demodulation is used for this purposed. I/Q demodulation was selected for the speed and simplicity of the algorithm, requiring only two multiplications over the set of input measurements, along with a subsequent filter step.

**Fig. 9:**
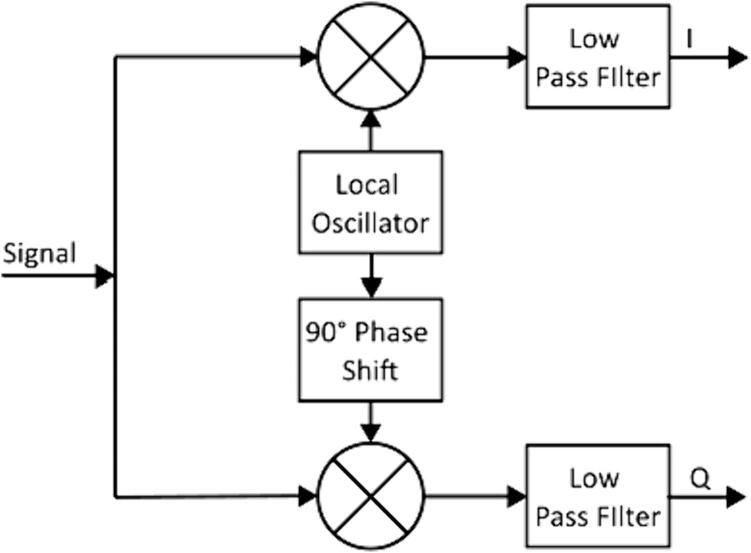
Block diagram of I/Q demodulator.

The sampled signal is transformed into components known as the in-phase and quadrature components by multiplying it separately by two sine waves – one in phase with the original excitation, and one shifted by 90°. This process is illustrated in Fig. 9. A low pass filter is also applied to each signal. In this case, to further reduce complexity, a simple mean was applied in place of a more complex filter algorithm.

The *I* and *Q* signals can then be treated as a complex number. The continuous signal magnitude is the magnitude of this complex number. The continuous signal phase is its phase, resulting in the following equations for signal magnitude (M) and phase (Φ):

(12) $$M=\sqrt{I^{2}+Q^{2}}$$

(13) $$\Phi =\tan^{-1}\left(\frac{Q}{I}\right)$$

To minimize error in the I/Q demodulation process, it is important to select an appropriate ratio between the signal frequency and the sampling frequency in I/Q demodulation. Error is minimal when the sampling period divides a whole number of times into the total sampling time (signal period times number of sampled cycles).

### Data Format

Table 4 shows the format of the ASCII message frame used to transmit one frame of EIT measurements.

**Table 4:** EIT data format.

Frame start	Measurement 1	Measurement separator 1	Measurement n	Measurement separator n	Frame end
**14 Characters**	**Variable characters (float, 4dp)**	**2 characters**	**Variable characters (float, 4dp)**	**2 characters**	**2 characters**
“\r\nmagnitudes:”	e.g,: “1.1234”	“,”	e.g,: “1.1234”	“,”	“\r\n”

Note: • “n” refers to the number of measurements in a frame. It varies depending on the dist_exc and step_meas parameters (It is 192 for the default parameter settings). • The number of characters in each measurement can vary depending on the value of the measurement.

### User Interface

The EIT system provides two methods of user interface: a text-based serial interface and a graphical application. The graphical application is open source and is provided in supplementary material under EIT_Data_Acquisition.zip.

### Serial Interface

**Fig. 10:**
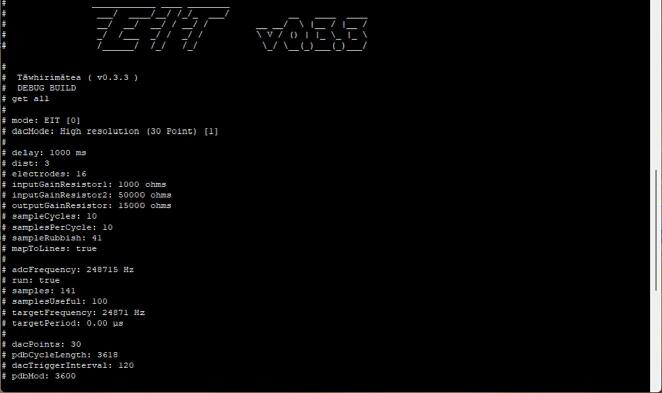
Screenshot of EIT serial interface.

A view of the serial interface is shown in Fig. 10. The EIT serial interface can be accessed using a desktop serial client such as PuTTY [26]. In this interface, settings on the electronics module can be modified using ASCII encoded commands. It is also one method to view the continuous streaming measurements from the EIT module.

### Graphical Interface

**Fig. 11:**
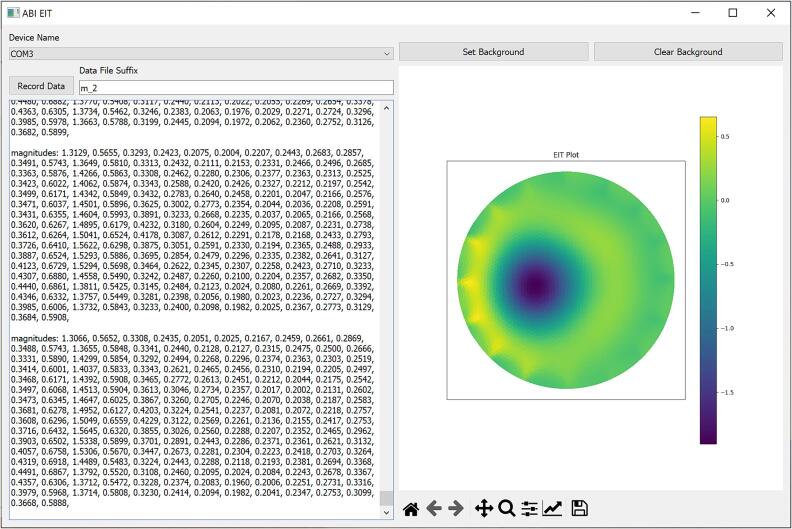
Screenshot of graphical EIT application.

The second user interface option for the EIT system is the graphical application. A screenshot is shown in Fig. 11. The graphical application connects to the electronics module via USB and performs real-time reconstructions of the EIT data using the open source pyEIT library. The reconstruction mesh and parameters can be modified using a configuration file. The application also provides the ability to write the streaming EIT data to a file.

### Design Files

The electronics module schematics and PCB diagram are available in both Altium Designer format and.PDF. The electronics module is described by the schematic files shown in Table 5.

**Table 5:** Summary of design files.

Document Name	Contents
EIT_Teensy_PCBMain.PcbDoc	PCB layout
EIT_Teensy.SchDoc	Top level connections between component documents
EIT_Teensy_VCCS.SchDoc	Voltage controlled current source
EIT_Teensy_Teensy3.6.SchDoc	Connections to Teensy I/O pins
EIT_Teensy_Amplifiers.SchDoc	Measurement amplifiers
EIT_Teensy_DDS.SchDoc	DDS module
EIT_Teensy_Mux.SchDoc	Multiplexers
EIT_Teensy_PSU.SchDoc	Power supply unit
EIT_Teensy_Connections.SchDoc	Electrode connection header

### Bill of Materials

The bill of materials is provided in supplementary material. It contains details of the components required to assemble the electronics module PCB, as exported from the Altium project.

### Build Instructions

1. The electronics module can be purchased pre-assembled from a PCB assembly manufacturer such as PCBWay. The required computer aided manufacturing (CAM) files and bill of materials are provided in supplementary material under EIT-Device-Electronics\EIT_Teensybasedv1\Generated Outputs\EIT_Teensy_PCBMainRev0.1_Gerbers_2021-05–25
2. Install the firmware on the Teensy microcontroller using the official Teensy Loader application. This simply requires a PC and a USB connection to the Teensy. The precompiled firmware in.HEX format is available in supplementary material.
3. Install the EIT app on a PC or tablet computer using the commands provided in the software repository.

### Operation Instructions

1. Connect EIT electronics module to the imaging domain (human subject or phantom) via the electrode connection header.
2. Connect EIT electronics module to the table computer via a USB cable.
3. Open the EIT app and select the appropriate COM port of the electronics module from the Device Name dropdown menu. Data will begin streaming to the app.
4. Use the Set Background button to set the current measurement frame as the background for time difference EIT reconstruction.
5. Use the Record Data button to record streaming data to a file.

### Validation and Characterization

The VCCS and Voltage Measurement components of the EIT electronics module were calibrated to improve and characterize their accuracy. For both components, we implemented a calibration protocol based on the ISO 11095 standard for linear calibration using reference materials. Once the system was calibrated, its performance was evaluated using the measures proposed in *Evaluation of EIT System Performance* [21]. These measures were implemented in python and contributed to the pyEIT project.

### Current Control Calibration

**Fig. 12:**
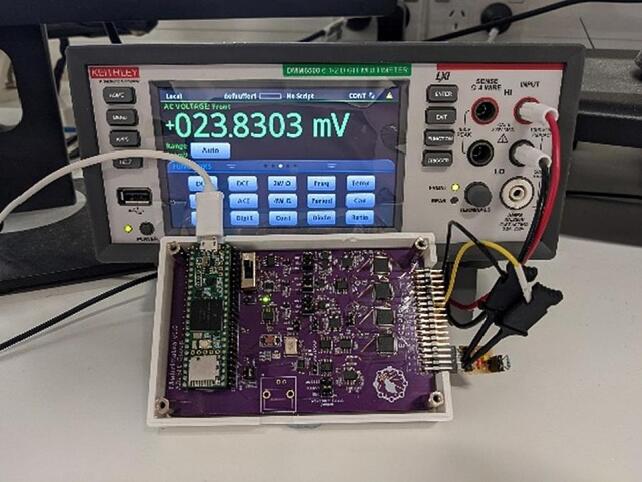
VCCS calibration setup showing EIT electronics module and Keithly multimeter.

The VCCS was calibrated using a protocol adapted from ISO 11095. The standard specifies a method for establishing a linear function which relates the values indicated by a measurement system and the corresponding accepted values of some reference materials. This function can then be used to make transformations of future measurements, resulting in an accurate estimation of the measured quantity. In the case of the VCCS, this method was used to establish the relationship between the current control set point and the actual current generated by the system, allowing us to construct a calibration function to estimate the set point required to achieve any given output current. In our formulation, the VCCS set point was analogous to the reference quantity, and an external measurement made of the output current was analogous to the value indicated by the measurement system. Output current was determined by measuring the voltage generated across a known load resistance on the output of the VCCS. A Keithly DMM6500 benchtop multimeter was used to take the voltage measurements as well as to accurately determine the load resistance. Fig. 12 shows the EIT electronics module with a discrete load resistor connected to the Keithly multimeter.

The performance of the VCCS is expected to be affected to some degree by the magnitude of the resistive load that the current is applied across. The calibration was therefore carried out at a range of test loads. To estimate a range of loads likely to be encountered when imaging the lungs of human subjects, we aimed to match measurement levels to data collected previously from a human subject (in a study approved by the Auckland Health Research Ethics Committee, AH252). We found that a load of approximately 60 Ω resulted in voltage levels matching data from a 30-year-old male subject of height 178 cm and weight 77 kg. Further test loads were selected at levels of 0.1, 0.5, 2, 10, and 100 times the 60 Ω point (translated into common resistor values). The parameters for the VCCS calibration protocol are shown in Table 6.

**Table 6:** VCCS Calibration Protocol.

Test Loads (Nominal)	6, 30, 57, 120, 560, 5600 Ω
Number of Repetitions	5
Test Currents (Set Point)	15, 20, 30, 40, 50, 60, 70, 80, 90, 100 µA RMS

ISO 11,095 provides the following model for a linear calibration function:

(14) $$y_{\mathrm{nk}}=\beta_{0}+\beta_{1}x_{n}+\varepsilon_{\mathrm{nk}}$$

In our formulation:

**Table:** 

$x_{n}$	Is the *n^th^* current set point.
$y_{\mathrm{nk}}$	Is the *k^th^* measurement of the current output under the *n^th^* set point.
$\beta_{0}+\beta_{1}x_{n}$	Represents the expected value of the measurements under the *n^th^* set point.
$\varepsilon_{\mathrm{nk}}$	Is the deviation between $y_{\mathrm{nk}}$ and the expected value of the measurements from the *n^th^* set point, assumed to be normally distributed with a constant variance $\sigma^{2}$.

Note that the model in equation (14) relies on the assumption that the standard deviations of repeated measurements at each of the reference points are equal, i.e., not proportional to the reference value. This is referred to as the assumption of constant residual standard deviation.

**Fig. 13:**
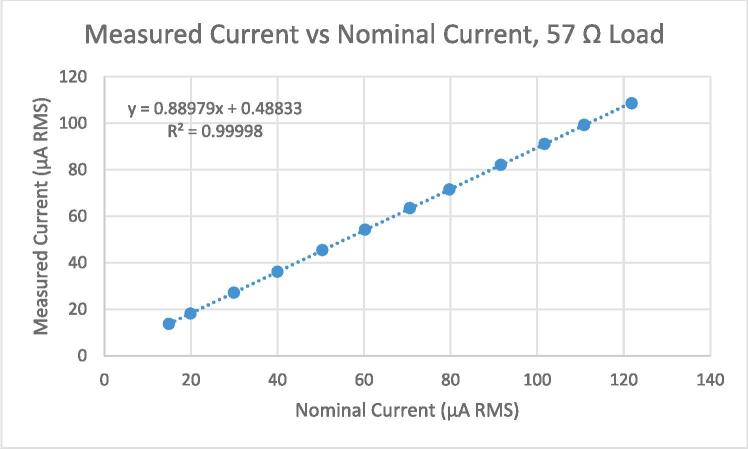
Measured current vs nominal current, using a load level of 57 Ω.

Fig. 13 shows the data collected for the 57 Ω load level.

As per the procedure defined in the standard, a linear regression was run to estimate the parameters of equation (14), i.e., to estimate $\beta_{0}$, $\beta_{1}$, and σ. For the 57 Ω load level, the results were the following: ${\hat{\beta }}_{1}$: 0.8899, ${\hat{\beta }}_{0}$: 0.4883 µA, $\hat{\sigma }$: 0.1381 µA. Using a threshold of 2 $\hat{\sigma }$, we interpret the uncertainty of the measurement (when calibrated with the calculated intercept and slope) to be ± 0.28 µA, at 95 % confidence. Table 7 shows a summary of the regression statistics for all six of the test loads.

**Table 7:** Summary of regression statistics for all test loads.

Load (Ω)	${\hat{\beta }}_{1}$	${\hat{\beta }}_{0}$(µA)	$\hat{\sigma }$(µA)
6.5	0.7483	4.8528	0.6147
30.8	0.8820	0.5335	0.1248
56.7	0.8898	0.4883	0.1248
120.8	0.8950	0.5112	0.1383
559.2	0.8689	0.4396	0.1533
5592	0.5195	0.2832	0.1024

**Fig. 14:**
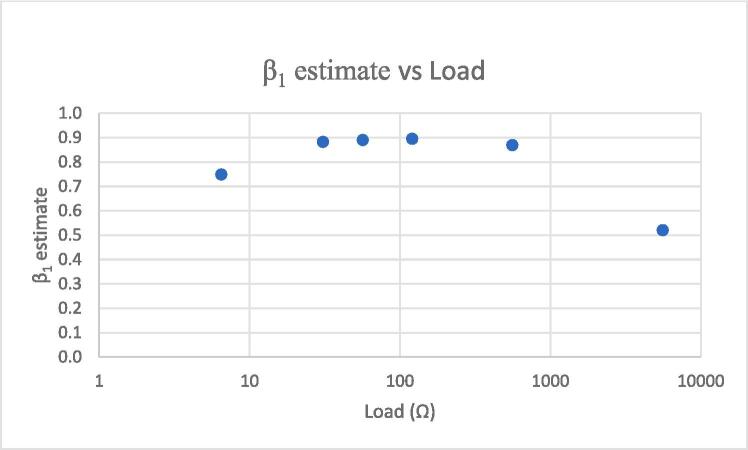
VCCS linear fit ${\hat{\beta }}_{1}$ vs load.

The estimates of the $\beta_{1}$ parameter for each load level are plotted in Fig. 14, demonstrating that VCCS performance is affected by load level, particularly strongly at the 6 Ω and 5,600 Ω levels.

**Fig. 15:**
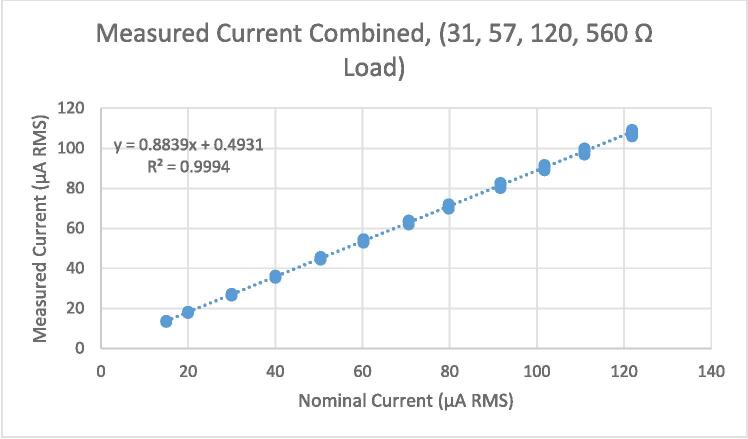
Measured vs nominal current for multiple load levels (31 Ω, 57 Ω, 120 Ω, 560 Ω).

In practice, it is not possible to know ahead of time what the load level will be. Therefore, it is necessary to find a single calibration that works best for a range of load levels. We calibrated for the range of 30 Ω to 560 Ω. Therefore, the current control accuracy for subjects who present a load level below or above this range will not be as good. Fig. 15 shows a plot of measured vs nominal current for this range.

**Fig. 16:**
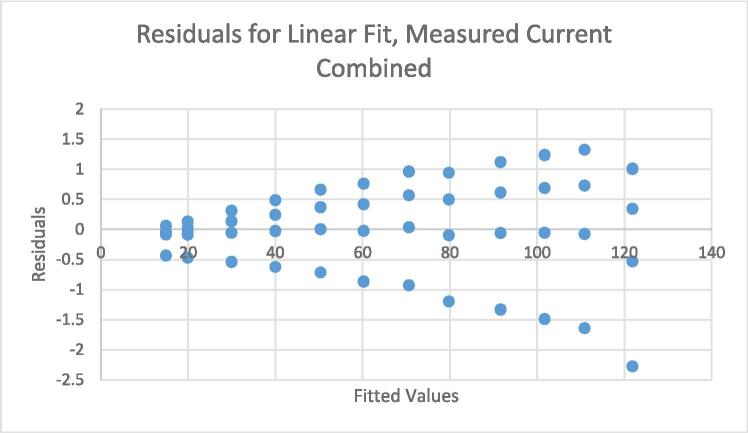
Residuals for linear fit of measured vs nominal current, multiple load levels.

Fig. 16 shows the residuals associated with the linear fit displayed in Fig. 15. It is clear that the dispersion of the residuals increases with the magnitude of the fitted values. Therefore, the assumption made in equation (14) of constant residual variance is violated. As per the procedure defined in the standard, this can be remedied by constructing a new model with the alternative assumption that residual standard deviation increases with reference value.

(15) $$y_{\mathrm{nk}}=y_{0}+y_{1}x_{n}+\eta_{\mathrm{nk}}$$

where

**Table:** 

$x_{n}$	Is the *n^th^* current set point.
$y_{\mathrm{nk}}$	Is the *k^th^* measurement of the current output under the *n^th^* set point.
$y_{0}+y_{1}x_{n}$	Represents the expected value of the measurements under the *n^th^* set point.
$\eta_{\mathrm{nk}}$	Is the deviation between $y_{\mathrm{nk}}$ and the expected value of the measurements from the *n^th^* set point. These deviations are assumed to be normally distributed with a variance proportional to $x_{n}^{2}$.

This model can be transformed in to one equivalent to equation (15) (i.e., with constant residual standard deviation) by dividing both sides of the equation by $x_{n}$, yielding:

(16) $$\frac{y_{\mathrm{nk}}}{x_{n}}=\frac{y_{0}}{x_{n}}+y_{1}+\frac{\eta_{\mathrm{nk}}}{x_{n}}$$

We can then estimate the parameters of equation (15) by running a regular (non-weighted) regression analysis on equation (16). Note that the slope for equation (15) will be the intercept for equation (16), and vice versa.

**Fig. 17:**
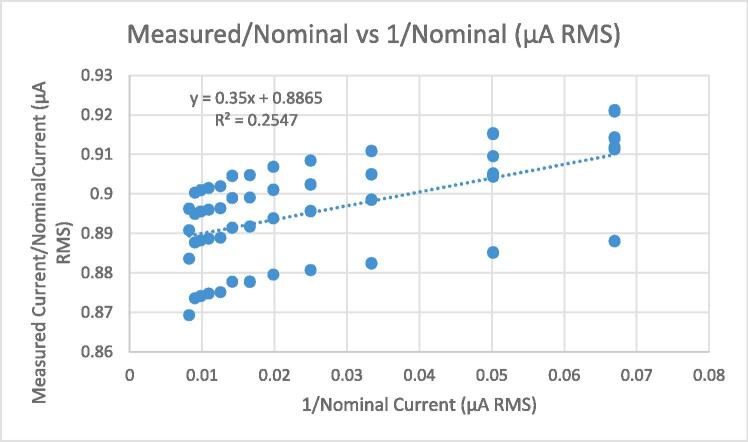
Measured/nominal current vs 1/nominal current.

The statistics for the regression analysis shown in Fig. 17 are as follows: Standard error: 0.0106 µA, Intercept: 0.8865 µA, Slope: 0.35. We interpret the uncertainty of the calibration in this case as ± 2.13 % of the measurement, at 95 % confidence.

The calibration function is therefore constructed as:

(17) $$x_{0}^{*}=\frac{{\overset{¯}{y}}_{0}-{\hat{y}}_{0}}{{\hat{y}}_{1}}$$

where

**Table:** 

$x_{0}^{*}$	Is the estimate of the required current set point.
${\overset{¯}{y}}_{0}$	Is the desired mean current output.
${\hat{y}}_{0},{\hat{y}}_{1}$	Are estimates of the parameters of equation (15).

Note that the 2 % uncertainty calculated for the combined range of load levels refers to the absolute accuracy in µA when the load level is allowed to vary from 31 Ω to 560 Ω. Over the course of all excitation pairs for a single subject, the load is unlikely to vary that much, and the variation in current output would be expected to be on the order of that calculated for the 57 Ω case.

### Voltage Measurement Calibration

The voltage measurement component of the EIT electronics module was also calibrated using a protocol adapted from ISO 11095. Individual calibration functions were constructed for three stages in the voltage measurement process: the two amplifier stages, and the conversion from analogue signal to the device’s reported voltage. Subsequently, a combined calibration function was constructed. In our formulation of the calibration procedure, the reference values were obtained by measurements made externally of the voltages at the input to the EIT device, and after the two amplifier stages. These measurements were made using the Keithly DMM6500 multimeter. The values corresponding to measurement system in the standard were obtained, in the case of the two amplifier stages, by dividing the externally measured output of each amplifier stage by its respective nominal gain, giving an estimate of the input to each stage. In the case of the analogue to digital conversion stage, the actual device reported voltage was used.

The input voltages were specified in terms of “equivalent load levels”. The equivalent voltage for a given load in ohms was defined as the voltage expected to be measured by the first pair of measurement electrodes in a measurement frame given that the load experienced by the excitation electrode pair was the specified resistance. Because of its placement close to the driving electrodes, the first measurement pair observes the highest voltage in a given frame, and thus our gain tuning protocol is to adjust the measurement gains to amplify this voltage to close to the full scale of the ADC. We therefore adjusted the two gain stages for each of test voltage levels to achieve this same effect. The parameters for this protocol are listed in Table 8.

**Table 8:** Voltage Measurement Calibration Protocol.

Equivalent Load	6 Ω to 600 Ω, 20 divisions evenly spaced on a log scale.
Applied Voltage	0.2 mV to 20 mV
Repetitions	5

The linear models for the three stages, Input Voltage to G1 Voltage, G1 Voltage to G2 Voltage, and G2 Voltage to Device Reported Voltage respectively are:

(18) $$\frac{w}{G_{1}}=w_{0}+w_{1}v+\varepsilon_{w}$$

(19) $$\frac{x}{G_{2}}=x_{0}+x_{1}w+\varepsilon_{x}$$

(20) $$y=y_{0}+y_{1}x+\varepsilon_{y}$$

where

**Table:** 

v,x,w	Are the mean voltages measured using the DMM6500 at the EIT device input, the output of the first amplifier stage, and the output of the second amplifier stage respectively.
$G_{1},G_{2}$	Are the nominal gains for the first and second amplifier stages respectively.
$\frac{w}{G_{1}},\frac{x}{G_{2}}$	Are uncalibrated estimates of v and w respectively.
y	Is the EIT device reported voltage.
$w_{0},x_{0},y_{0}$	Are the parameters defining the y-intercepts of the three linear models.
$w_{1,}x_{1},y_{1}$	Are the parameters defining the slopes of the three linear models.
$\varepsilon_{w},\varepsilon_{x},\varepsilon_{y}$	Are the parameters defining the variability between the models and the measured data.

As per the procedure defined in the standard, a linear regression was run for each of the three stages to estimate the parameters of equations (18),(19), and (20). The results for each stage are shown in Table 9.

**Table 9:** Calibration slope and intercept for three calibration stages.

Stage	Slope	Intercept (V)
Input Voltage to G1 Voltage	0.7873	−0.0002
G1 Voltage to G2 Voltage	0.989	−0.021
G2 Voltage to Device Reported Voltage	0.9897	−0.0006

We then combine the three stages to form a single model relating device reported voltage to input voltage:

$$y=y_{0}+y_{1}\left(G_{2}x_{0}+G_{2}x_{1}\left({G_{1}w}_{0}+{G_{1}w}_{1}v+{G_{1}\varepsilon }_{w}\right)+G_{2}\varepsilon_{x}\right)+\varepsilon_{y}$$

(21) $$y=y_{0}+y_{1}G_{2}x_{0}+{G_{1}G}_{2}{{y_{1}x}_{1}w}_{0}+{{G_{1}G}_{2}y}_{1}x_{1}w_{1}v+\varepsilon_{\mathrm{total}}$$

where

**Table:** 

$y_{0}+y_{1}G_{2}x_{0}+{G_{1}G}_{2}{{y_{1}x}_{1}w}_{0}$	Is the combined y-intercept.
${{G_{1}G}_{2}y}_{1}x_{1}w_{1}$	Is the combined slope.
$\varepsilon_{\mathrm{total}}$	Is the deviation between the expected value of device reported voltage at a given input voltage and the measured value.

And we construct the calibration function:

(22) $$v^{*}=\frac{\overset{¯}{y}-\left({\hat{y}}_{0}+{\hat{y}}_{1}G_{2}{\hat{x}}_{0}+{G_{1}G}_{2}{{{\hat{y}}_{1}\hat{x}}_{1}\hat{w}}_{0}\right)}{{{G_{1}G}_{2}\hat{y}}_{1}{\hat{x}}_{1}{\hat{w}}_{1}}$$

where

**Table:** 

$v^{*}$	Is the estimate of the true input voltage.
$\overset{¯}{y}$	Is the mean device reported voltage.
${\hat{y}}_{0},{\hat{y}}_{1,}{\hat{x}}_{0},{\hat{x}}_{1},{\hat{w}}_{0},{\hat{w}}_{1}$	Are estimates of the parameters of equation (22), given in
The linear models for the three stages, Input Voltage to G1 Voltage, G1 Voltage to G2 Voltage, and G2 Voltage to Device Reported Voltage respectively are:	
$\frac{w}{G_{1}}=w_{0}+w_{1}v+\varepsilon_{w},$	
(18)	
$\frac{x}{G_{2}}=x_{0}+x_{1}w+\varepsilon_{x}$	
(19)	
$y=y_{0}+y_{1}x+\varepsilon_{y}$	
(20)	
where	
v,x,w	Are the mean voltages measured using the DMM6500 at the EIT device input, the output of the first amplifier stage, and the output of the second amplifier stage respectively.
$G_{1},\;G_{2}$	Are the nominal gains for the first and second amplifier stages respectively.
$\frac{w}{G_{1}},\frac{x}{G_{2}}$	Are uncalibrated estimates of v and w respectively.
y	Is the EIT device reported voltage.
$w_{0},\;x_{0},\;y_{0}$	Are the parameters defining the y-intercepts of the three linear models.
$w_{1,}\;x_{1},y_{1}$	Are the parameters defining the slopes of the three linear models.
$\varepsilon_{w},\;\varepsilon_{x},\;\varepsilon_{y}$	Are the parameters defining the variability between the models and the measured data.
As per the procedure defined in the standard, a linear regression was run for each of the three stages to estimate the parameters of equations (18),(19), and (20). The results for each stage are shown in Table 9.	
Table 9.	
${G_{1},G}_{2}$	Are the nominal gains for the two amplifier stages.

Equation (22) can also be used to calculate appropriate amplifier gain values based on the expected input voltage and the desired device reported voltage. Since there are two variables to set, an optimization algorithm must be used to select gain levels that satisfy both equation (22) and the available gain settings as controlled by the measurement circuit digital potentiometers.

**Fig. 18:**
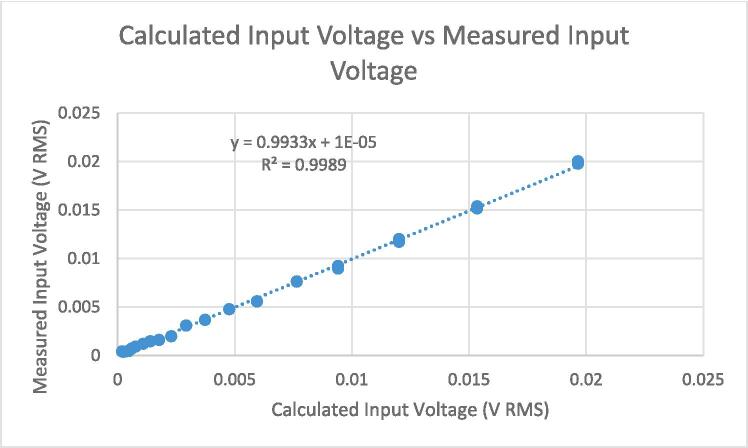
Calculated input voltage vs measured input voltage.

Fig. 18 shows input voltage calculated by applying the calibration function described by equation (22) vs measured input voltage. By running a regression analysis, we compute an uncertainty of − 0.7 % ± 0.36 mV, at 95 % confidence.

### EIT System Performance

The performance of the system for EIT reconstruction was measured using data experimentally gathered from a salt bath type phantom. The test protocol and performance measures were adapted from *Evaluation of EIT system performance* [21]. All performance measures were implemented in python and contributed to pyEIT.

**Fig. 19:**
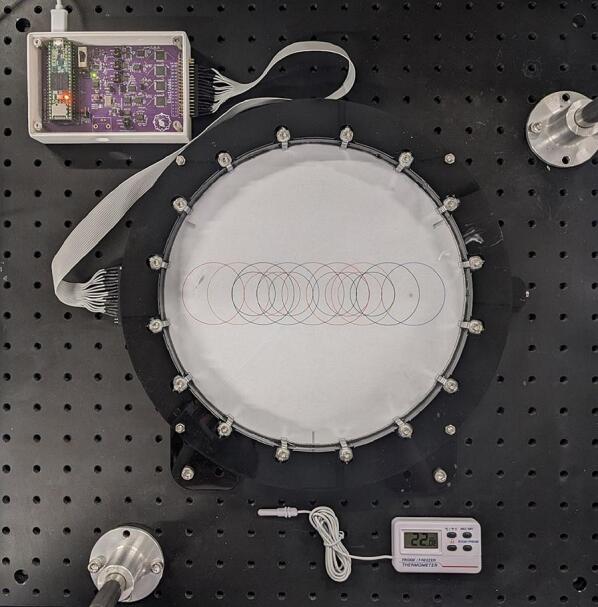
Salt bath phantom.

The phantom used is shown in Fig. 19. It consists of an acrylic bath 240 mm in diameter, with 16 stainless steel electrodes of 4.6 mm width equally spaced around the perimeter. The bath was filled to a depth of 10 mm with a solution of 36.7 g/L table salt (approx. 99 % NaCl) in water. This concentration was selected by modelling the resistance between two electrodes separated by 67.5° (i.e., a distance of 3 apart), and choosing the conductivity which resulting in a measurement of 64 Ω (0.194 Ωm). The relationship between conductivity and the concentration of our solution was calculated using the model given in [27].

A printed template was placed under the transparent bottom of the bath, allowing us to position targets in the bath accurately by hand.

### Measurements

The following sets of measurements were taken:

**Empty Bath Measurements**: Measurements were taken with the bath filled with solution, but empty of any targets. 20 frames were taken at approximately 2 frames per second, which were used to compute the signal to noise ratio and accuracy measures, as well as for the background of the difference EIT reconstructions involving the single target measurements. After completing the remaining measurements, data was collected for at least 2 h at approximately 1 frame per second for the purposes of the Allan deviation and drift measures.

**Single Target Measurements**: A circular acrylic target of 50 mm diameter was placed in the bath at 9 positions equally spaced 20 mm apart, as indicated in the printed template by the large circles. 20 frames of data were collected at each position. A period of at least 30 s was observed at each position before beginning to collect data in order to wait for movement of the solution to subside.

**Fig. 20:**
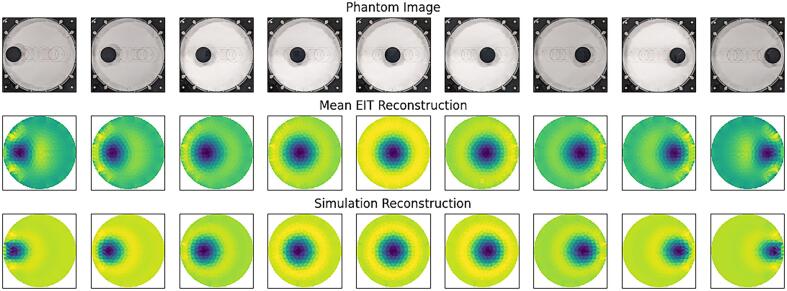
Single target test – phantom photograph, reconstructed images, and simulated images.

Fig. 20 shows an image of the target in each of the 9 positions, along with corresponding EIT reconstructions and simulated reconstructions. EIT reconstruction was performed using a linear time difference reconstruction algorithm provided by the pyEIT JAC class, implemented as:

(23) $$\hat{x}={\left(J^{T}J+\lambda R\right)}^{-1}J^{T}y$$

(24) $$R={\left[J^{T}J\right]}_{i,i}^{p}$$

where

**Table:** 

$\hat{x}$	Is an estimate of the conductivity values of the elements in the imaging domain.
y	Is the difference between two sets of measured voltages.
J	Is the Jacobian matrix.
λ	Is the regularization hyperparameter that controls the degree of regularization.
R	Is the regularization matrix, made up of diagonal elements of $J^{T}J$, scaled by the exponent p.

The hyperparameter λ was set to 0.05 heuristically to improve shape reconstruction and reduce ringing, and the *p* parameter was set to the default 0.5, a compromise between pushing noise to the boundary and to the centre, as discussed in [28].

**Fig. 21:**
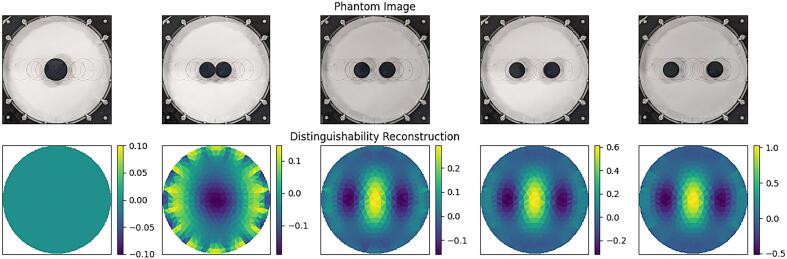
Double target test – phantom photograph and reconstructed images.

**Two Target Measurements:** Two circular acrylic targets of half the area of the larger target (i.e., 35.36 mm diameter) were placed in the bath in 4 different positions, each increasing in separation distance by 20 mm. As with the single target measurements, a period of at least 30 s was observed before collecting 20 frames of data. Fig. 21 shows an image of each of the positions of the two-target measurement set. See *distinguishability* below for discussion of the performance measures associated with this test. Note the rising scale in the last three reconstruction images, correlated with rising distinguishability.

**Frame Processing Speed Measurements:** In addition to the single target positional measurements, a series of measurements was taken with the 50 mm target at a position of *r*/0.5 while progressively decreasing the number of cycles sampled, and thus increasing the frame processing speed. The number of sample cycles used was 10, 8, 6, 4, 2, and 1.

### Performance Measures

Using the data collected as described above, the following performance measures were computed. These are described in more detail in [21]. The last four are also known as GREIT figures of merit[29]:

**Signal to noise ratio:** The measurement of signal to noise ratio is computed from the empty bath data, with a value for each of the 192 individual measurements in the EIT data frame (for our measurement protocol), described as “channels” in [21]. The signal to noise ratio is the ratio of the mean of each channel to its standard deviation. This is expressed in decibels as a root-power quantity by taking the log_10_ of the ratio and multiplying by 20.

**Accuracy:** Accuracy is computed as the closeness of the mean background measurement for each channel to a simulated measurement, after each data set has been normalized to lie between 0 and 1.

**Allan Deviation:** Allan deviation is a calculation commonly used to measure frequency stability, which in this case is used to indicate the presence of measurement drift over a period of time. Deviations are computed over an increasing range of periods and should trend downwards if no drift is present. Note: this is referred to as “Drift” in [21], but we have also calculated drift in a more traditional manner.

**Drift:** Drift is a measure of the shift in value of a measurement over a period of time. It is simply calculated as the difference in mean values measured at specific points in time.

**Detectability**: Detectability is a measure used to compare the ability to reliably detect targets at different points within the measurement domain. It is calculated as the ratio between the mean value of reconstructed image pixels in a given region of interest, and the standard deviation of those pixels. The region of interest is defined using a threshold process, where pixels of value greater than one fourth of the maximum pixel value in the image are selected. This is expressed in decibels as a root-power quantity by taking the log_10_ of the ratio and multiplying by 20.

**Distinguishability:** Distinguishability is a measure of the ability to distinguish structural details of objects, for example, to distinguish between one target and two of the same combined area. It is calculated using the same algorithm as detectability, but instead of an empty background, a frame containing the object from which subsequent targets are to be distinguished is used. (See Fig. 21).

**Amplitude:** Amplitude is a measure of the average value of pixels in the reconstruction image.

**Position Error:** Position error is a measure of the error between the position of an object in the reconstruction image and that of the object in a simulated reconstruction image. It is calculated as the difference between the two distances from the centre of mass of the image.

**Resolution:** Resolution is a measure of the size of the reconstructed target as a fraction of the total area of the medium. It is calculated as the square root of the target area over the medium area.

**Ringing:** The effect known as “Ringing” is an image reconstruction artifact where pixels of opposite value to the target appear outside the region of interest. The Ringing measure is the ratio of the total amplitude of these pixels to those inside the region of interest.

## Results

**Fig. 22:**
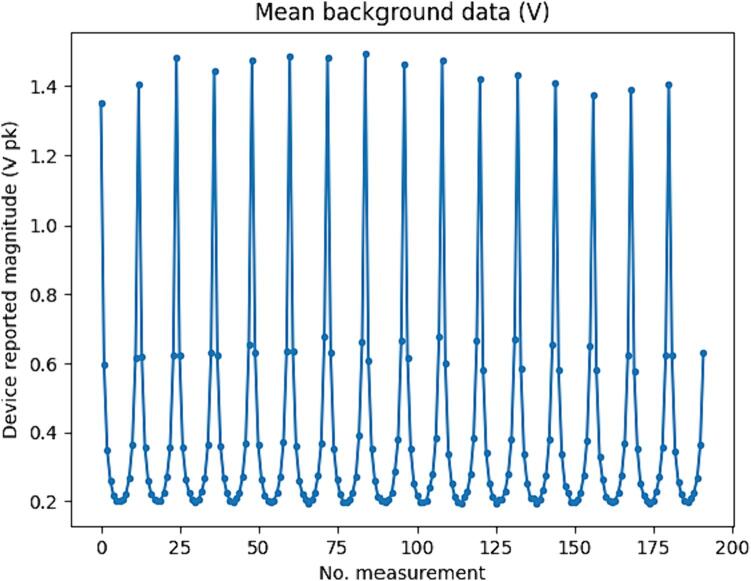
Mean empty bath measurements.

Fig. 22 shows the mean measurements for all 192 channels averaged over 20 frames taken with an empty bath. The maximum channel mean was 1.5 V (Amplitude, i.e., peak of the sinusoidal waveform), and the minimum was 0.19 V (peak). The full-scale voltage of the ADC is 3.3 V, so the total measurement gain was roughly 90 % of the maximum possible for this setup. Using the reported effective resolution of the ADC of 13 bit, the LSB is 0.4 mV, giving a quantization error of the minimum measurements of 0.2 %.

**Fig. 23:**
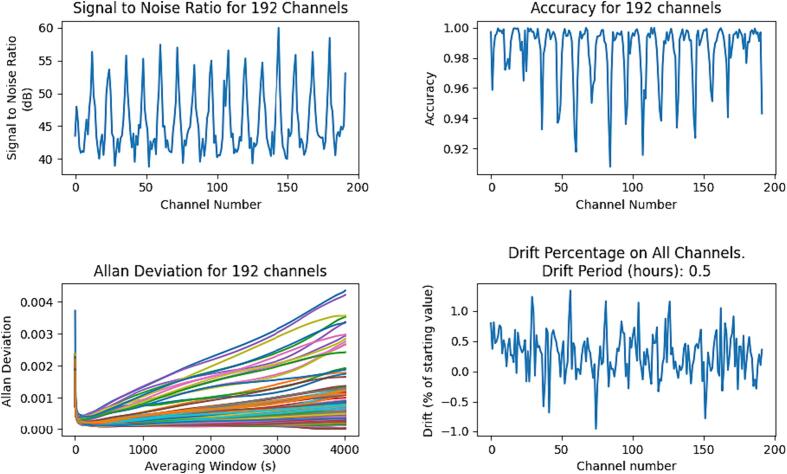
Signal to noise ratio, accuracy, Allan deviation, and drift.

Fig. 23 shows signal to noise ratio, accuracy, Allan deviation, and drift. Signal to noise ratio and accuracy are calculated for each channel using the 20 empty bath background frames. The signal to noise ratio is between 36 dB and 63 dB, and the minimum accuracy is 91 %.

The Allan deviation is plotted for each channel with windows between 0 s and 4000 s. Considerable drift is evident. However, the mean drift over a period of 30  min is only around 0.25 %.

**Fig. 24:**
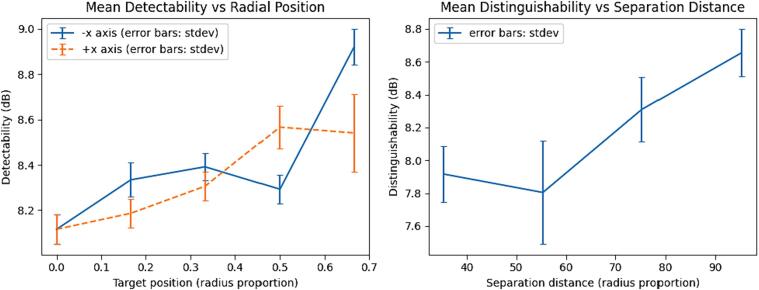
Detectability and distinguishability.

Fig. 24 shows detectability and distinguishability. Detectability increases with radial position of the target. Distinguishability increases with separation distance.

**Fig. 25:**
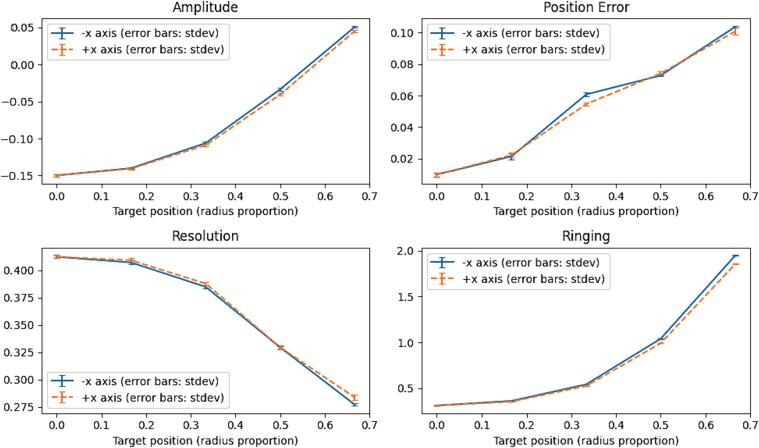
Amplitude, position error, resolution, and ringing.

Fig. 25 shows amplitude, position error, resolution, and ringing plotted against radial position, in both the negative and positive directions. For all measures, the negative and positive sides tend to agree well. Amplitude, position error, and ringing all increase with radial position, while resolution decreases.

**Fig. 26:**
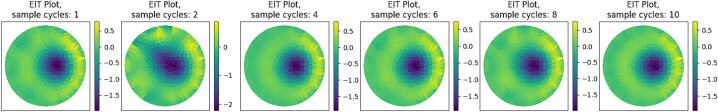
Varying sample cycle reconstructions.

**Fig. 27:**
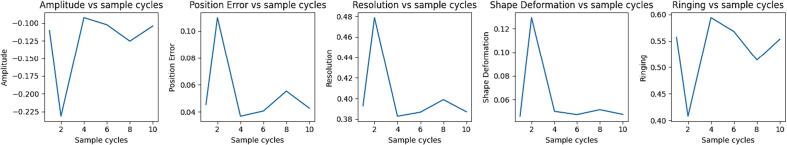
GREIT figures of merit vs sample cycles.

Fig. 26 shows the EIT reconstructions for each of the varying sample cycle measurements. For each of the measurements, the GREIT figures of merit were calculated, shown in Fig. 27. Except for an anomaly at 2 sample cycles, there was not much difference in the reconstruction quality between 10 and 1 sample cycles. The frame processing frequency at 1 sample cycle is 24.8 Hz. The normal human respiratory frequency is about 0.2 Hz [30]. Therefore, by the Nyquist-Shannon sampling theorem [31], the frame processing frequency of the EIT device is high enough to completely determine the components of the respiratory signal up to 60 times the base frequency.

## Conclusions

We have developed a wearable, low-cost EIT system suitable for scanning the lungs of free-breathing subjects. All schematics, designs, and source code has been published under an open-source license. Its design is similar to many other devices in the literature, but to our knowledge is the first complete open-source device suitable for imaging free breathing subjects. Our device has been designed to meet the safety requirements of the IEC 60601 standard, adherence to which is surprisingly uncommon among EIT devices published by research groups.

The voltage measurement accuracy of our device has been measured to be − 0.7 % ± 0.36 mV, at 95 % confidence. While existing literature does not commonly include accuracy measurements in these terms, this number will be useful in assessing future device improvements, and in creating accurate EIT simulations. If this performance measure becomes more common in the EIT literature, it could also aid in assessing the contributions to application specific performance from intrinsic device characteristics compared with those from experimental setup or reconstruction algorithm selection.

Our device has also been tested against a set of performance measures adapted from *Yasin et al.* in *Evaluation of EIT system performance* [21]*.* Of these measures, our device performed similarly to theirs in signal to noise ratio and accuracy. Drift, at 0.25 % in 30 min was held to be acceptable. Amplitude, position error, resolution and ringing all indicated good symmetry in the EIT system, and displayed similar trends to those observed by *Yasin et al.* Our device performed worse in detectability and distinguishability than theirs, but still indicated significant observable signal in an experimental setup designed to be suitable for our intended application.

While the hardware design of our device is intended to be suitable for imaging free breathing subjects, we have yet to study its performance *in vivo.* The usability of the device is also limited by the open-source software that it relies on, lacking, for example, lung specific functional EIT measures. Future studies should implement these metrics in the pyEIT software package and measure the device’s performance in human subjects.

It is our hope that this work will contribute to an increased consensus in EIT hardware design and testing, as well as increasing the accessibility to low-cost EIT hardware to other research groups. Our group plans to use this device for further EIT study both in phantoms and in human subjects.

Andrew Creegan is a Doctoral Candidate at the Auckland Bioengineering Institute. His research area is Electrical Impedance Tomography, with a particular focus on lung diseases such as pulmonary oedema.

## CRediT authorship contribution statement

**Andrew Creegan:** Writing – review & editing, Writing – original draft, Software, Investigation, Formal analysis. **Joshua Bradfield:** Writing – review & editing, Software, Investigation. **Samuel Richardson:** Writing – review & editing, Investigation. **Llewellyn Sims Johns:** Writing – review & editing, Investigation. **Kelly Burrowes:** Project administration, Funding acquisition. **Haribalan Kumar:** Project administration, Funding acquisition. **Poul M.F. Nielsen:** Project administration, Funding acquisition. **Merryn H. Tawhai:** Project administration, Funding acquisition.

## Declaration of competing interest

The authors declare that they have no known competing financial interests or personal relationships that could have appeared to influence the work reported in this paper.
